# Environmental Salinity Modifies Mucus Exudation and Energy Use in European Sea Bass Juveniles

**DOI:** 10.3390/ani11061580

**Published:** 2021-05-28

**Authors:** Borja Ordóñez-Grande, Pedro M. Guerreiro, Ignasi Sanahuja, Laura Fernández-Alacid, Antoni Ibarz

**Affiliations:** 1 Department of Cell Biology, Physiology and Immunology, University of Barcelona (UB), 08028 Barcelona, Spain; b.ordonez@ub.edu (B.O.-G.); isanahuja@ub.edu (I.S.); tibarz@ub.edu (A.I.); 2 CCMAR—Centre for Marine Sciences, University of Algarve, 8005-139 Faro, Portugal; pmgg@ualg.pt

**Keywords:** *Dicentrarchus labrax*, mucus exudation, salinity adaptation, osmoregulation, gill Na^+^/K^+^-ATPase

## Abstract

**Simple Summary:**

Changes in skin mucus production and composition offer a new means to study how fish cope with changes in the environment. We explored the utility of skin mucus as an indicator of physiological responses and energy use in a reference fish species, the European sea bass. We evaluated the exudation volume of skin mucus and the main stress- and osmoregulation-related biomarkers in both mucus and plasma. We demonstrate the viability to study the exuded volume of skin mucus composition and its parameters as an informative tool of the fish energy waste at different environmental salinities. This study is of great interest for both aquaculture and ecological studies.

**Abstract:**

The European sea bass (*Dicentrarchus labrax*) is a euryhaline marine teleost that can often be found in brackish and freshwater or even in hypersaline environments. Here, we exposed sea bass juveniles to sustained salinity challenges for 15 days, simulating one hypoosmotic (3‰), one isosmotic (12‰) and one hyperosmotic (50‰) environment, in addition to control (35‰). We analyzed parameters of skin mucus exudation and mucus biomarkers, as a minimally invasive tool, and plasma biomarkers. Additionally, Na^+^/K^+^-ATPase activity was measured, as well as the gill mucous cell distribution, type and shape. The volume of exuded mucus increased significantly under all the salinity challenges, increasing by 130% at 50‰ condition. Significantly greater amounts of soluble protein (3.9 ± 0.6 mg at 50‰ vs. 1.1 ± 0.2 mg at 35‰, *p* < 0.05) and lactate (4.0 ± 1.0 µg at 50‰ vs. 1.2 ± 0.3 µg at 35‰, *p* < 0.05) were released, with clear energy expenditure. Gill ATPase activity was significantly higher at the extreme salinities, and the gill mucous cell distribution was rearranged, with more acid and neutral mucin mucous cells at 50‰. Skin mucus osmolality suggested an osmoregulatory function as an ion-trap layer in hypoosmotic conditions, retaining osmosis-related ions. Overall, when sea bass cope with different salinities, the hyperosmotic condition (50‰) demanded more energy than the extreme hypoosmotic condition.

## 1. Introduction

Wild European sea bass (*Dicentrarchus labrax*) moves seasonally from seawater to freshwater environments and vice versa, including coastal areas, lagoons, estuaries and other parts of rivers [1,2,3,4]. Despite this haline plasticity, water salinity can affect sea bass growth in extreme conditions below 10‰ and over 50‰, as already reported by Dendrinos and Thorpe [5] and Eroldogan et al. [6] who found better growth performance at lower salinities (10‰, 20‰, 25‰ and 30‰) than control (33‰). Varsamos et al. [1,2] measured blood osmolality at larval and juvenile stages, while Jensen et al. [7] studied the effect of salinity on osmoregulation and branchial Na^+^/K^+^-ATPase. In those studies, the authors suggested that the acclimation process was completed in four to eight days. Similar responses were found when analyzing growth performance, osmoregulatory and metabolism as part of the acclimation process in other marine species, such as gilthead sea bream (*Sparus aurata*) [8,9,10,11,12], shi drum (*Umbrina cirrosa*) [13] and red porgy (*Pagrus pagrus*) [14]. Within the first few days, a classic pattern develops known as “crisis and regulation”, which consists of an initial phase of blood metabolic and osmotic changes, followed by a phase of regulation, when osmoregulatory and metabolic parameters achieve a steady “normalized” state [3,7,9,13]. The first evidence of this is a variation of blood osmolality and the main osmosis-related ions (Na^+^, Cl^−^ and K^+^) [7,11,12,14,15,16]. Gills are markedly affected by these osmotic changes, which modify Na^+^/K^+^-ATPase activity and chloride cell dynamics processes which are mediated by cortisol [17,18,19,20]. This eventually restructures gill energy metabolism and requirements [8,10,11,21,22]. Meanwhile, blood metabolic changes are mainly related to a decrease in plasma glucose, triglycerides and cholesterol [3,7,9,13]. After a condition has been sustained for weeks, energy metabolism is reorganized towards an increased energy expenditure, reallocation of resources and depletion of carbohydrate reserves in several tissues, such as liver, gills, kidney and brain [8,9,10,12,14].

Although blood analysis is a non-lethal method to measure stress, the procedure can result in injuries on blood vessels, leading to hemorrhage, in fish skin, which may increase the risk of infection. Therefore, in recent years, growing interest has been shown in the use of minimally-invasive methods to assess fish physiological status and welfare, such as fish skin mucus analysis [23,24,25,26,27]. It has also been reported that endogenous and exogenous factors, such as fish developmental stage, sex, infections or environmental changes, can modify fish skin mucus composition [23,24,25,26,28,29,30,31,32]. Moreover, it has been observed that the components of exuded mucus are modified in response to stressors [33,34,35,36,37], including acute salinity challenges [27]. Indeed, measurement of some stress indicators found in mucus, such as cortisol, glucose and lactate, has been proposed as a feasible, non-invasive, analysis of stress biomarkers [23,24,25,38,39,40]. Nonetheless, to date, most experiments have analyzed short-term stress, with few long-term studies. Recently, Fernández-Montero et al. [41] studied the effect of different stressors, such as temperature, stock density and handling, on cortisol release and mucins expression in the skin of the greater amberjack *Seriola dumerili*. They reported an increase in *muc*-2 expression, which encodes a secreted glycoprotein forming part of the insoluble mucous barrier, in high stock densities and in response to handling protocols, indicating a possible increase in mucus exudation in relation to stress. In addition, skin mucus has many relevant biological and ecological roles, and among them osmoregulation [42,43]. Previously Roberts and Powell [44] evaluated skin mucus modifications under different salinities over three months in salmonids, finding that skin mucus was hyperosmotic with regard to hypoosmotic surrounding water, and that gill mucous cells shifted from neutral to acid when fish were moved from freshwater to seawater. Recently, we used skin mucus biomarkers to evaluate the response of sea bass to several acute osmotic challenges [27] and observed, under hypersalinity conditions, the production of a very large volume of skin mucus, with the highest total contents of cortisol, glucose, and protein. Thus, although that study only focused on the acute response, this could be an undesirable condition if the salinity condition becomes chronic.

Considering the above-mentioned studies, we believe that changes in skin mucus production and composition offer a new means to study how fish cope with changes in the surrounding water. Despite a number of papers focused on fish osmoregulatory responses there is less information on the metabolic impacts of salinity adaptation, and little research has considered skin mucus as a target or indicator for the study of osmotic response. Thus, our main aim here was to study skin mucus biomarkers, together with plasma and gill parameters, in the response of juvenile sea bass to sustained osmotic challenges. To this end, we exposed seawater (35‰) acclimated fish for 15 days to a hypersaline condition (50‰) and to two hyposaline environments: An almost freshwater condition (3‰) and a mid-estuary condition (12‰), which is practically isoosmotic to the fish internal milieu. We explored the usefulness of mucus as an indicator of physiological responses and energy usage by evaluating the volume of mucus exuded and the main stress-related and osmoregulation-related biomarkers in mucus and plasma. Moreover, we also analyzed gill energy usage (Na^+^/K^+^-ATPase activity) and gill mucous cell classes and shapes. All these findings contribute to increased knowledge about the acclimation responses of European sea bass to environmental salinity and the repercussions in the energy expenditure required to maintain homeostasis under a chronic condition, which could be useful for conservation biology and aquaculture.

## 2. Materials and Methods

### 2.1. The Animals and Experimental Procedures

European sea bass juveniles were obtained from a commercial source (Mariscos de Esteros, SA, Huelva, Spain) and acclimated indoors at the CCMAR Ramalhete marine station (Faro, Portugal). Fish were reared for two months in open-system fiberglass tanks (1000 L), at seawater salinity (34.9‰ ± 0.1‰) pumped from the marine environment at the naturally occurring temperature (15.7 °C ± 0.2 °C ) under the natural photoperiod (April to May, 2019), and fed a commercial diet twice a day (2.5% *w*/*w*). For the assay, fish (129.2 ± 3.6 g) were randomly allocated into 500 L tanks (*n* = 10/tank at 2–3 Kg/m^3^) in 4 semi-closed systems. After one week, fish were acclimated to experimental conditions aiming at water salinities of 3‰, 12‰, 35‰ and 50‰. The transition between 35‰ and each experimental condition was carried out during three days by increasing freshwater flow in the systems for the 3‰ and 12‰ conditions, and by adding highly concentrated seawater (prepared with commercial sea salt) in the system for the 50‰ condition. Once the experimental salinities were achieved, the fish were kept in the experimental tanks for a period of 15 days and fed normally until 24 h prior to sampling. To maintain low ammonia levels and assure good water quality, the systems were not completely closed. In control and low salinities, the salinities were achieved by balancing the flow of seawater, SW, with that of freshwater, FW (so 100% SW for 35‰, 35% SW + 65% FW for 12‰ and 8.5% SW + 91.5% FW for 3‰). These mixtures were prepared in a head tank and flowed into the fish tanks. For the high salinity, 50‰, sea salt was added and mixed in the head tank and flowed into the fish tank and recycled into the head tank. Renewals of the water in the head tank were performed every 48 h. Oxygenation and adequate mixing were provided by strong aeration in the head tanks and oxygenation was also guaranteed by aeration in the fish tanks. Water salinity, oxygen saturation and ammonia levels were controlled throughout this period. Measured salinities varied by 0.5 to 1 ppt above and below the target values; oxygen saturation values were always above 85% and ammonia levels were always below 2.5 mg/L. The 15-day exposure period was selected in accordance with the reported effects of osmotic challenges on sea bass osmoregulation [7,8,9,11,12,45,46].

After the 15-day period, the animals from each condition were sampled for skin mucus, blood and gills. All fish from each tank were sampled before moving to the next condition in a rapid procedure that did not expand more than 30 min per tank. Individual mucus samples were collected once the fish was anaesthetized with 2-phenoxyethanol (1:250, Sigma-Aldrich, Madrid, Spain) as described in Fernández-Alacid et al. [23]. The fish removal from the tank and its onset of sedation took less than 1 min, and no fish remained more than 5 min under sedation before the mucus sampling. A sterile glass slide was used to carefully remove mucus from the over-lateral line, starting from the front and sliding in the caudal direction. The glass was gently slid along both sides of the animal, avoiding the non-desirable operculum, ventral-anal and caudal fin areas, and the skin mucus was carefully collected into a sterile tube (1.5 mL), snap-frozen in dry ice and stored at −80 °C until analysis. Thereafter, each fish was laterally (all on the left side) photographed with a Nikon D3000 camera (Nikon, Tokyo, Japan), weighed and measured. Blood was subsequently obtained from the caudal vein with a 1 mL heparinized syringe with a 23G needle. Plasma was separated from whole blood by centrifugation at 10,000× *g* for 5 min, aliquoted, immediately frozen and stored at −80 °C. The animals were then killed by severing the spinal cord and gill filaments from the second gill arch was collected and placed in a tube with 100 μL of ice-cold SEI (sucrose-EDTA-imidazole) buffer (150 mM sucrose, 10 mM EDTA, 50 mM imidazole, pH 7.3) [47], snap-frozen and stored at −80 °C. An additional section from the same gill arch was severed and placed in a 2 mL tube with Bouin-Holland solution.

The research was approved by the Centre for Marine Sciences (CCMAR)-Universidade do Algarve animal welfare body (ORBEA) and the Direção-Geral de Alimentação e Veterinária (DGAV), Permit 2019-06-04-009758, in accordance with the requirements imposed by Directive 2010/63/EU of the European Parliament and of the Council of 22 September 2010 on the protection of animals used for scientific purposes.

### 2.2. Metabolite Biomarkers and Cortisol Levels in Mucus and Plasma

The soluble components of skin mucus samples were previously obtained from the mucus homogenization, using a sterile Teflon pestle and centrifugation at 14,000× *g* for 15 min [23]. Enzymatic colorimetric tests for glucose and lactate (LO-POD glucose and LO-POD lactate, SPINREACT, Sant Esteve de Bas, Spain) adapted to 96-well microplates and fish mucus and plasma samples were used. Following the manufacturer’s instructions, mucus and plasma samples and standard dilutions were mixed with working reagents in triplicate. The OD was determined at 505 nm with a microplate reader (Infinity Pro200 spectrophotometer, Tecan, Barcelona, Spain). The glucose and lactate values were expressed as mg·dL^−1^ for plasma and μg·mL^−1^ for skin mucus.

Cortisol levels were measured using an ELISA kit (IBL International, Hamburg, Germany). Briefly, an unknown amount of antigen present in the sample competed with a fixed amount of enzyme-labelled antigen for the binding sites of the antibodies coated onto wells. After incubation, the wells were washed to halt the competition reaction. Therefore, after the substrate reaction, the intensity of the color was inversely proportional to the amount of antigen in the sample. Following the manufacturer’s instructions and adaptations for fish mucus and plasma [24,25], the samples and standard dilutions (from 0 to 3 μg·dL^−1^) were mixed with enzyme conjugate and incubated for 2 h at room temperature. The substrate solution was added after rinsing the wells with a wash solution and incubated for 30 min. The reaction was halted by adding stop solution and the OD was determined at 450 nm with a microplate reader (Infinity Pro200 spectrophotometer, Tecan). The cortisol values were expressed as ng cortisol·mL^−1^ of plasma or skin mucus.

Mucus and plasma protein concentrations, as well as protein in gill homogenates (for Na^+^/K^+^-ATPase activity, see ahead), were determined using the Bradford assay [48] with bovine serum albumin (BSA) as the standard. Bradford reagent was mixed with the samples in triplicate and incubated for 5 min at room temperature. The OD was determined at 595 nm with a microplate reader (Infinity Pro200 spectrophotometer, Tecan or MultiScanGo, ThremoFisher Scientific, Rochester, NY, USA). The protein values in plasma or skin mucus were expressed as mg protein·mL^−1^.

### 2.3. Osmolality and Ion Quantification of Plasma and Skin Mucus

Plasma osmolality was measured with a vapor pressure osmometer (Wescor Vapro 5520, ELITechGroup, Norwood, MA, USA) and was expressed as mOsmol·kg^−1^. Plasma Na^+^ and K^+^ levels were measured using a Flame Photometer (BWB XP, BWB Technologies, Berkshire, UK) and expressed as mmol·L^−1^. Plasma chloride concentration was measured using a colorimetric test (SPINREACT, Spain) adapted to microplates, and the OD was determined in a microplate reader (MultiScanGo, ThremoFisher Scientific); measured values were expressed as mmol·L^−1^. Mucus osmolality and ion concentrations (Na^+^, K^+^ and Cl^−^) were measured using an ion analyzer (ISElyte X9, Tecil, Barcelona, Spain). Osmolality values were expressed as mOsmol·kg^−1^ and ion concentrations as mmol·L^−1^.

### 2.4. Mucus Exudation Values

To determine the effects of the osmotic challenge, total mucus exudation was obtained by measuring the volume of mucus collected (in μL), related to the skin area (in cm^2^) and to fish weight (in g). Skin area was obtained using the ImageJ program (US National Institutes of Health, Bethesda, MD, USA). The area was manually marked as an approximation of the area actually scrapped, avoiding the dorsal and the lateral fins, and over the lateral line. This was then measured using the software included in the program. Furthermore, soluble collected mucus (μL) was referred to the sampling area and to fish weight, to calculate collected mucus per area (μL·cm^−2^) and collected mucus per unit weight (μL·g^−1^).

### 2.5. Gill Na^+^/K^+^-ATPase Activity

Gill Na^+^/K^+^-ATPase activity was determined using the method developed by McCormick [47] adapted for microplate assay [49]. Gill tissue was homogenized in 125 µL of SEI buffer with 0.1% of deoxycholic acid and centrifuged at 2000× *g* for 30 s. Samples were mixed with the assay buffer with or without 0.5 mM ouabain and a decrease in absorbance was measured at 340 nm for 15 min at 25 °C (MultiScanGo, Thermo Scientific, Rochester, NY, USA). An enzymatic coupling of ATP dephosphorylation to NADH oxidation was used to detect ouabain-sensitive ATPase activity, and Na^+^/K^+^-ATPase activity was expressed as μmol ADP·mg protein^−1^·h^−1^. Protein in gill homogenates was determined as indicated previously.

### 2.6. Gill Histology and Histological Analysis

After 24 h in Bouin solution at RT, the gill arches were rinsed several times in 70% ethanol and stored at 4 °C. The tissues were cleared in graded xylene and were later embedded in paraffin (Paraplast Plus; Sherwood Medical, St Louis, MO, USA) and sectioned at 6 μm. After dewaxing and rehydration, the sections were placed on slides with APES treatment (Aminopropyltriethoxysilane, Sigma, Madrid, Spain). The slides were stained using a periodic acid-Schiff (PAS) and Alcian Blue (AB) staining protocol. For histological analysis, the slides were photographed using a light microscope (BX61; Olympus, Tokyo, Japan) connected to a digital camera (DP70; Olympus, Tokyo, Japan) at a magnification of ×20. Goblet cells were counted on 6 no-consecutive lamellae sections of 250 µm, from the second branchial arch, of 5 fish per condition. The counts were performed in blind condition with sections from all samplings mixed and counted by one person. Goblet cells were counted using ImageJ (US National Institutes of Health, Bethesda, MD, USA), while cell counting, frequency (cell·mm^−2^), size (μm^2^), perimeter (μm) and shape were calculated, with acid mucins (purple) and neutral mucins (magenta) differentiation.

### 2.7. Statistical Analyses

The experiment was performed without tank replicates and a priori statistical evaluation was performed to obtain the minimal number of fish required for a one-way ANOVA, based on fixed effects. To compare the data obtained for stress-related biomarkers, osmotic parameters and Na^+^/K^+^-ATPase activities for the different salinity challenges, we used one-way ANOVA. Additionally, Student’s t-test was used to compare osmotic parameters between plasma and mucus. For all our statistical analysis, a priory study for homogeneity of variance was performed using Levene’s test. When homogeneity existed, Tuckey’s test was applied; if homogeneity did not exist, then the T3-Dunnet test was applied. All statistical analysis was undertaken using SPSS Statistics for Windows, Version 22.0 (IBM Corp, Armonk, NY, USA) and all differences were considered statistically significant at *p* < 0.05.

## 3. Results

Body weight, body length and condition factor were obtained, and no significant differences were observed in response to the osmotic challenges (Table 1). To determine the effects of sustained salinity challenge on osmoregulatory homeostasis, plasma osmolality together with osmotic-related ions (Na^+^, Cl^−^ and K^+^) were measured (Table 2). Although plasma osmolality strongly buffered the changes in water salinity, maintenance for 15 days at 3‰ provoked a slight but significant reduction in plasma osmolality (322 ± 3 mOsmol·kg^−1^, *p* < 0.05) while the maintenance at 50‰ significantly increased plasma osmolality by 5% (358 ± 5 mOsmol·kg^−1^, *p* < 0.05). The sum of the two major main osmosis-related ions, Na^+^-Cl^−^, represented around 90% of plasma osmolality, irrespective of the challenge condition, even if levels at 50‰ (but not at low salinities) were significantly elevated in relation to control. Interestingly, plasma potassium was lower (*p* < 0.05) than control at both hypo- and hyperosmotic challenges.

### 3.1. Gill Na^+^/K^+^-ATPase Activity and Gill Mucous Cells

**Figure 1:**
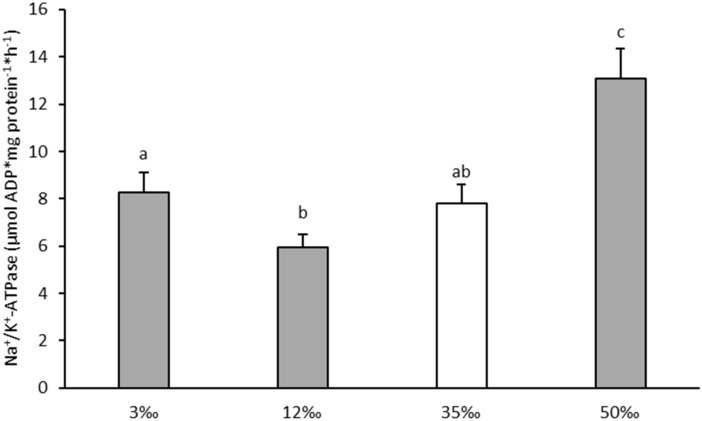
Gill Na^+^/K^+^-ATPase activity of European sea bass juveniles in response to a chronic osmotic challenge after 15 days. Values are shown as mean ± standard error of mean. *n* = 10. Letters indicate significant differences among salinities challenges (*p* < 0.05, ANOVA and post-hoc Tuckey test). The value of 35‰ (white bar) is assumed as control value of seawater salinity.

**Figure 2:**
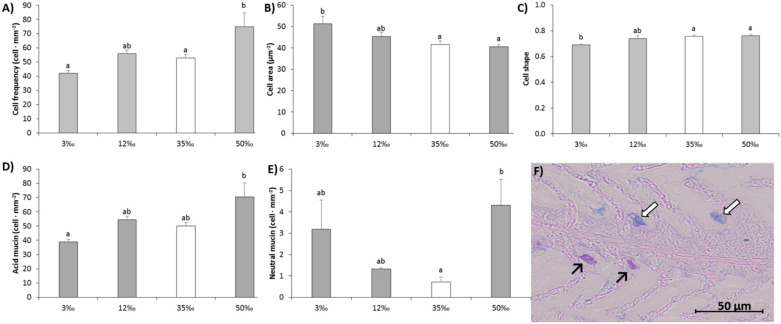
Gill mucous cell count of cell frequency (**A**), size (**B**) and shape (**C**) and gill mucous cell count of acid (**D**) and neutral (**E**) mucins of European sea bass juveniles in response to a chronic osmotic challenge. Image of histological differentiation of acid and neutral mucins (**F**). Scale bar: 0.5 μm. Values are shown as mean ± standard error of mean. *n* = 10. Different letters indicate different groups of significance among salinities challenges (3‰, 12‰, 35‰ and 50 ‰) by one-way ANOVA analysis and post-hoc Tuckey’s test (*p* < 0.05). The value of 35‰ (white bar) is assumed as control value of seawater salinity.

At 50‰, Na^+^/K^+^-ATPase activity was significantly increased (by around 60%, *p* < 0.05) in relation to control values at 35‰ (Figure 1). Although Na^+^/K^+^-ATPase activity at the lower salinities did not differ significantly from control, values in fish at 12‰ (a near isosmotic condition to plasma osmolality) were the lowest and significantly below those recorded for fish at 3‰ (*p* < 0.05). The distribution of gill mucous cells (Figure 2) was markedly affected by the salinity challenges, which impacted cell frequency, size and shape (where the shape value = 1 corresponded to perfect circular cell). Cell counts showed that acclimation to hypersalinity induced a significant proliferation of mucous cells. Higher number of cells per mm^2^ of gill filament were detected (*p* < 0.05) at 50‰ than at 35‰ (Figure 2A), showing greater abundance of both types of mucous cells, acid (white arrows) and neutrals (black arrows) (Figure 2D,E), but with no differences in cell size (Figure 2B) or shape (Figure 2C). At lower salinities; however, acclimation to 12‰ condition did not modify any mucous cell parameters (with regard to control values), exposure to 3‰ resulted in the smaller number of cells per filament and significant changes in cell size (increased area) and shape (more elongated). Overall, gill acid mucin cells were far more numerous than neutral mucin cells, but the ratio between acid mucin cells and neutral mucins cells, which is indicative of gill mucus composition, was modified by the extreme conditions: for fish acclimated to the intermediate salinities this ratio was approximately 47:1 and 45:1 at 35‰ and 12‰ respectively, for fish in either hypo-osmotic or hyperosmotic salinities the ratio was significantly reduced to 12:1 at 3‰ (*p* < 0.05) and 17:1 at 50‰ (*p* < 0.05).

### 3.2. Mucus Exudation Parameters

**Figure 3:**
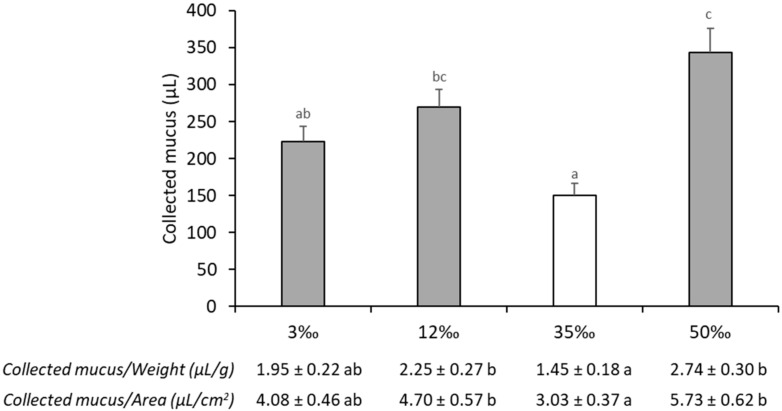
Skin mucus exudation parameters of European sea bass submitted to a chronic osmotic challenge. Values are shown as mean ± standard error of mean. *n* = 10. Letters indicate significant differences among salinities challenges (*p* < 0.05, ANOVA and post-hoc Tuckey test). 35‰ (white bar) is assumed as control value of seawater salinity.

The skin mucus volume was recorded (Figure 3) and the exuded volume per area of collection and per unit of body weight were calculated (included in Figure 3). The amount of skin mucus increased in all conditions in relation to control (*p* < 0.05). In both hyposaline environments, 3‰ and 12‰, the volume of mucus exuded was increased by 50% and 80% in relation to those at 35‰, at Day 15 of exposition, and this trend remained when the exuded mucus was expressed per area of collection or per unit of body weight. The hyperosmotic challenge at 50‰ provoked the greatest skin mucus over-exudation, 130% (*p* < 0.05) of the control values at 35‰, which meant that the mucus exudation per cm^2^ of the calculated fish surface increased from 3.03 ± 0.37 µL at 35‰ to 5.73 ± 0.62 µL at 50‰ (*p* < 0.05).

### 3.3. Plasma and Mucus Osmoregulation

**Figure 4:**
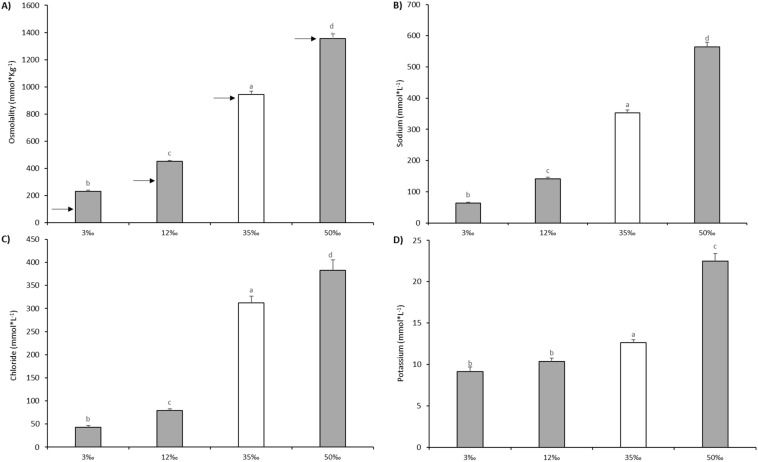
Skin mucus osmolality (**A**) and main osmotic-related ions (**B**–**D**) of European sea bass submitted to a chronic osmotic challenge. Values are shown as mean ± standard error of mean. *n* = 10. (**A**) Black arrows correspond to measured osmolality of surrounding water (3‰ = 115 mOsmol·kg^−1^, at 12‰ = 320 mOsmol·kg^−1^, at 35‰ = 931 mOsmol·kg^−1^, at 50‰ = 1366 mOsmol·kg^−1^). Different letters indicate different groups of significance among salinities challenges (3‰, 12‰, 35‰ and 50 ‰) by one-way ANOVA analysis and post-hoc Tuckey’s test (*p* < 0.05). 35‰ (white bar) is assumed as control value of seawater salinity.

Compared with plasma, mucus osmolality did not buffer water salinity, and was strongly correlated with environmental osmolality (Figure 4A). Mucus osmolality at 35‰ was 944 ± 24 mOsmol·kg^−1^, almost identical to the values measured in water and quite different from the plasma control values (339 ± 5 mOsmol·kg^−1^). A similar situation was observed at 50‰ but in lower salinities mucus osmolality was maintained at values 40% and 100% above water osmolality (for fish at 12‰ and 3‰ respectively), which may be related to the presence of organic molecules as osmolytes or to increased ion retention capacity of mucus at low salinities. Ions (Na^+^, Cl^−^ and K^+^) were measured in skin mucus (Figure 4B–D). Interestingly, the sum of main the osmosis-related ions, Na^+^ and Cl^−^, in mucus proved to be a better approach to water osmolality than the whole mucus osmolality (101.5 ± 5.6 mmol·L^−1^ for 3‰ and 225.2 ± 6.9 mmol·L^−1^ for 12‰). In fact, the Na^+^-Cl^−^ sum explained 36.6% ± 2.5% and 50.2% ± 1.4%, respectively, of the total measured mucus osmolality (Figure 4). Consequently, other mucus components must contribute greatly to mucus osmolality. On the contrary, the 35‰ and 50‰ conditions showed closer osmolarities between mucus and water, and the Na^+^-Cl^−^ sum covered around 64.02% ± 1.94% and 70.14% ± 1.19%, respectively, but far from the 90% covered in plasma. Interestingly, mucus K^+^ concentration, although not participating greatly in total osmolality values, did not show the same proportions for the conditions and mucus potassium showed the higher values at 50‰ (*p* < 0.05).

### 3.4. Physiological Biomarkers in Mucus and Plasma

**Figure 5:**
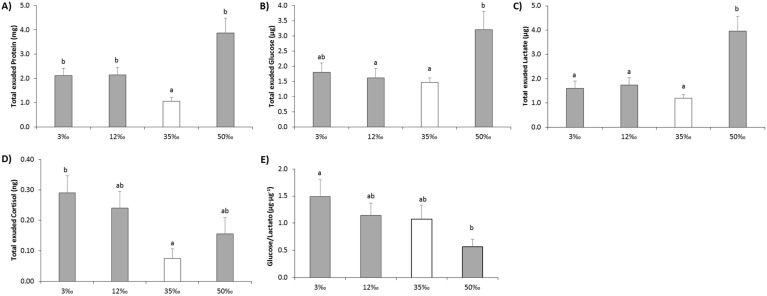
Total exuded biomarkers (**A**–**D**) and glucose:lactate ratio (**E**) in skin mucus of European sea bass juveniles in response to a chronic osmotic challenge. Values are shown as mean ± standard error of mean. *n* = 10. Different letters indicate different groups of significance among salinities challenges (3‰, 12‰, 35‰ and 50 ‰) by one-way ANOVA analysis and post-hoc Tuckey’s test (*p* < 0.05). The value of 35‰ (white bar) is assumed as control value of seawater salinity.

Main skin mucus biomarkers, such as soluble protein, glucose and lactate, as well as cortisol exuded levels exhibited different responses to the osmotic challenges (Table 3). In response to the extreme salinities of 3‰ and 50‰, significant increases in protein exudation were recorded: from 6.12 ± 0.61 mg·mL^−1^ for control mucus to 9.44 ± 0.85 mg·mL^−1^ (> 50% higher, *p* < 0.05) and to 10.81 ± 1.05 mg·mL^−1^ (> 75% higher, *p* < 0.05) for the 3‰ and 50‰ challenges, respectively (Figure 5A). Cortisol, one of the main indicators of acute stress response, appeared to be exuded in greater amounts (*p* < 0.05) in the lowest salinity, 3‰, while for both 12‰ and 50‰, mucus cortisol did not differ from control values. However, mucus glucose did not change significantly among the different salinities while mucus lactate was two-fold over-exuded at 50‰ compared to control. The same biomarkers were also measured in plasma. Protein levels in plasma showed the same trend as in skin mucus, with the lowest values observed in the 35‰ condition. However, significant differences in plasma protein occurred only in the 3‰ fish (which was 66.7% higher, *p* < 0.05). Glucose levels did not show any significant differences between the osmotic conditions, as reported in mucus. A significant increase in plasma lactate concentrations was observed in all challenging conditions in relation to control: Lactate was 80% higher in the 3‰ condition (*p* < 0.05) and 30% higher in both 12‰ and 50‰ conditions (*p* < 0.05). In contrast, plasma cortisol concentration was highest at 35‰, being twofold (3‰ and 12‰ conditions) and threefold (50‰ condition) higher than at other salinities; however, it was only significantly different from the 50‰ condition (*p* < 0.05). No significant correlation, by the Pearson’s coefficients analyses, was found between skin mucus and plasma biomarkers.

### 3.5. Energy Expenditure by Mucus Exudation

As the individual volumes of mucus exuded were recorded, the total amount of each biomarker in mucus was calculated and showed in Figure 5, to evaluate their loss as future waste and energy expenditure. Thus, both hypoosmotic conditions caused significant over-exudation of protein (Figure 5A), glucose (Figure 5B) and lactate (Figure 5C) with respect to the control condition. The hyperosmotic condition generated the greatest and significantly highest release of the same metabolites, two-fold greater (*p* < 0.05) than the release at hypoosmotic conditions and five-fold with respect to control values (*p* < 0.05). This represents a sustained energy expenditure when animals are maintained at this salinity. With regard to mucus cortisol levels (Figure 5D), the 3‰ challenge resulted in a significant three-fold increase of total exuded cortisol, compared to control values. Lastly, the ratio of glucose:lactate exuded is also represented in Figure 5E, as an indicator of changes in aerobic metabolism in response to the salinity challenges. After 15 days at the experimental salinities, the mucus glucose:lactate ratio was significantly higher (*p* < 0.05) at 3‰ than at 50‰, evidencing different aerobic metabolism responses.

## 4. Discussion

European sea bass is an euryhaline marine teleost species that withstands different salinity conditions, hypoosmotic and hyperosmotic. Here we focused on evaluating the volume of mucus exuded as well as the amount of several different biomarkers contained in that mucus in response to sustained exposure to low (3‰ and 12‰) and high (50‰) salinity. We show that, in addition to osmoregulatory and metabolic changes, complete acclimation to such conditions has important impacts on mucus production and composition. Moreover, gill activity and modifications in gill mucous cell class, distribution and shape were recorded. All these data, together with parameters related to osmosis, allowed us to relate chronic salinity challenges with fish energy expenditure and waste via skin mucus over-exudation.

The total energy consumed by osmoregulatory adaptation was estimated to be between 20%–68% in different species [50], but these values must include not only the actual cost of ion transport but consider the energy used by other metabolic processes which respond to changes in salinity [51]. Salinity-triggered hormones affect different pathways of energetic metabolism and other non-osmoregulatory organs such as the liver and brain also show changes in energetic metabolism [9,12,14,52,53,54,55]. Nonetheless, it is generally expected that a reduced gradient between the environment and the internal milieu lowers the need for osmoregulatory activity allowing more energy to be used for other functions [50] but this appears to be a more complex, and multifactorial process. In fact, previous reports have indicated the optimal conditions for sea bass growth to be between the isosmotic salinities 12‰ and 15‰ [7,8,56,57,58], but additional studies also show that 28‰ to 30‰ were better growth conditions for sea bass [5,59]. In our study, no significant differences were found in morphometric parameters (fish growth, length or condition factor) between the experimental conditions after two weeks. In a previous study, we already noted the relevance of measuring exuded mucus volume under an acute salinity challenge for sea bass [27], which could be further related to energy costs to maintain increased mucus exudation over time. In the current experiment, we recorded the volume of mucus exuded in response to the proposed challenges. For all the conditions, the amount of skin mucus increased with respect to the control condition and was 130% higher for the hyperosmotic condition. It is well established that fish exude more mucus in stressful situations, as described in acute stress experiments [23,24,25,27]. Several studies have reported an apparent increase in mucus production when fish transition both from freshwater to seawater [60,61,62,63,64] and from seawater to freshwater [43,65,66,67]. Nonetheless, those empirical findings were not tested, as mucus exudation was not measured. Thus, for the first time, here we have provided data on the exacerbated and continued skin mucus exudation in response to salinity challenges in sea bass. In consequence, fish energy status could be affected due to the need to exude the many mucus components, such as the gel-forming mucins, which are heavy and large glycoproteins [41], or the large numbers of soluble proteins [37] and the energy metabolites, like glucose and lactate [23].

### 4.1. Plasma Changes, Gill Activity and Stress Impacts of Osmoregulatory Responses

Plasma osmolality has been used as a physiological indicator when measuring the effects of salinity on fish physiology [11,68,69,70,71]. Adult euryhaline teleosts maintain plasma osmolality between 300 and 350 mOsm·kg^−1^ under tolerable salinities [11,72,73]. However, this adjustment is not immediate, and unbalances may occur in the first hours or days post transfer indicating incomplete osmoregulation in plasma [7,74,75,76]. For instance, after abruptly transferring European sea bass to freshwater, Jensen et al. [7] recorded plasma hemodilution, with plasma osmolality at 240 mOsm·kg^−1^ and being tolerated for at least 10 days. In the present case, 15 days after the initial salinity change, European sea bass showed a slight but significant decrease in the hypoosmotic conditions and a slight but significant increase in the hyperosmotic condition, compared to the control condition. However, those changes in the average plasma osmolality were minimal and variance within each group were quite low which suggests that a new equilibrium was achieved (i.e., a compromise for favorable osmoregulation in each salinity). It seems therefore, from the current data, that sea bass acclimated well to a 15-day exposure to altered salinity, maintaining plasma osmolality within narrow homeostatic limits (11% variation range limited by a 6% increase and 5% decrease in average osmolality levels at the highest and lowest salinity conditions, respectively).

Changes in plasma osmolality were paralleled by variations in the concentrations of the major ions sodium and chloride, which, together with potassium, account for around 90% of the total osmolality. Plasma Na^+^ and Cl^−^ followed the trends in osmolality while K^+^ actually dropped in hypo- and hyper-osmotic condition in relation to fish at 35‰. The concentration of these ions in plasma is mostly controlled by the action of the branchial Na^+^/K^+^-ATPase, which has a paramount role in salinity acclimation favoring the excretion of Na^+^ and Cl^−^ in hyperosmotic conditions and being responsible for ion uptake in hypoosmotic conditions [18,77,78]. In the present study, Na^+^/K^+^-ATPase activity showed a typically “U-shape”, as reported before, in European sea bass [7,11,13], with the lowest activity being under the isosmotic condition (12‰) [2,7,11,13,50]. This shape of Na^+^/K^+^-ATPase activity has been associated to relevant changes in gill energy metabolism during adaptation to extreme salinity conditions, as 3‰ and 50‰, and could represent an increase in consumption of alternative energetic substrates such as lactate [9].

There is no doubt that salinity challenges, despite well accommodated, represent an energy-demanding stress. The most commonly used physiological stress indicators in fish are plasma hormones, namely cortisol, and metabolites [79,80]. Cortisol is the principal glucocorticoid secreted under conditions of acute stress, via stimulation of the neuroendocrine system hypothalamus-hypophysis-interrenal (HPI), and a posterior cascade of metabolic and physiological changes occurs, making glucose and lactate readily available to the tissues [81,82]. In most fish, both metabolites and cortisol reach their highest circulating concentrations within a few hours, with plasma levels being stressor dependent and species specific, and with greater discrepancies and controversy when the stressor is chronic [83,84]. In the current chronic challenges, it was unexpected to observe higher cortisol levels in the control group, but that had no correspondence with the downstream metabolites. Cortisol is also associated with the onset of hyposmoregulation, namely in salmonids and euryhaline fish [77,85,86], but this is again not clear in our data as fish in 50‰ showed the lowest cortisol values. As for other prolonged stressful situations, it is possible that the response of the HPI may be less sensitive. Further studies will be necessary to assess this concern in fish exposed to the more challenging situations [87].

When related to chronic salinity acclimation, plasma lactate appeared to be the most affected indicator. Usually plasma lactate tends to increase in situations of increased activity or reduced oxygen availability [82,87], but it has been observed in both hypoosmotic and hyperosmotic acclimated fish [88,89]. It could be considered that lactate becomes an important source of energy during osmotic acclimation as it can supply energy to different tissues, such as gills, kidney and brain as it was previously reported in several fish species [9,11,90,91,92]. For its part, plasma glucose concentration registered no significant differences due to salinity challenges, becoming a less discriminating metabolite during long-term osmotic acclimation, as also reported for other stressors [8,9,12,13]. No clear function for plasma protein has been suggested yet in long-term osmotic acclimations, but Sangiao-Alvarellos et al. [8] hypothesized that plasma protein functionality could be related to secondary metabolic reallocation of energy resources, once carbohydrate storage has been mobilized and exhausted. In our study, soluble protein is significantly changed only for the 3‰ condition, showing an increase. While some authors indicate that plasma protein increases as salinity changes [8], other authors report no changes [93] or even a reduction [94].

### 4.2. Gill Mucus Cells and Skin Mucus Changes in Response to Salinity

In this study, the impact of salinity on gill remodeling was analyzed histologically by total mucus cell counts and by measuring cell parameters, such as mucus cell class, frequency and shape. Overall, the frequency of mucus cells in gill filaments increased in fish exposed to higher salinity. Although we have not measured production, it is likely these cells were active, thus increasing the amount of mucus secretion in the hyperosmotic environment in relation to other conditions. Gill mucus was suggested to provide a selective semi-permeable layer during salinity change [95] and in the Japanese eel (*Anguilla japonica*), gill mucus cells were activated after SW transfer, producing Na-binding molecule-containing mucus layers that protect against the high osmolality of SW [96], while in the red sea bream (*Pagrus major*) the amount of secreted mucus showed a decreased trend when transferred to low salinity [97]. These previous observations are in agreement with our cell frequency data.

The nature of the mucins produced is also modified, and the mucus cells of shi drum (*Umbrina cirrosa*) reared at full seawater contained a mixture of acid and neutral mucins, whereas in fish adapted to hypo-osmotic environment only neutral mucins were observed [13]. In fact higher cell counts of neutral mucin mucus cells in freshwater and acidic mucin mucus cells in seawater have been recorded in different species, such as gilthead sea bream, rainbow trout [98], and shi drum [13]. In agreement with those results, we recorded a gradual increase in acid mucin mucus cells in response to salinity with the highest frequency at 50‰, which almost doubled the 3‰ cell frequency. Concomitantly, the frequency of neutral mucin mucus cells, which were much lower than for acid mucin mucus cells, increased in the lowest salinity. However, interestingly, the highest salinity and cell frequency was also over threefold greater in both extreme conditions than at 35‰.

With regard to the role of skin mucus, it is known to be involved in fish osmoregulation. In this line, discussing the role of mucus in freshwater fish, Shephard [42] mentioned the impermeability function of skin mucus in water and ion flux diffusion but suggested this function would only reduce water diffusion by 10%. Mucus (skin and gill) were also previously suggested to support active ion uptake by concentrating cations from an ion-deficient environment [99]. In the same way, skin mucus as a polyanionic gel, has the potential to trap cations and allow anion diffusion [100,101], thus reducing both ion gradients to the plasma and ion transport processes and thereby allowing for a reduction in the cost of ion transport [102]. In our observations, skin mucus maintained osmolarity identical to seawater at high salinities but above environmental water osmolality at low salinities, as previously reported for freshwater salmonids [44], thereby reinforcing its possible role as an ion capture mechanism [99]. In addition, we found that the mucus concentration of Na^+^ and Cl^−^ the main osmosis-related ions, represented a very low proportion of the total osmolality in the 3‰ and 12‰ conditions and that these ions were also slightly underrepresented in mucus at other salinity conditions in relation to seawater ion concentrations. This difference indicates that osmoregulation might be mediated by other molecules exuded in mucus at higher proportions at low salinities than under control conditions or high salinities, thereby preserving osmosis-related ions inside the body of the fish. Nevertheless, despite significant decreases in absolute values, mucus K^+^ concentration was relatively conserved at values similar to those of 35‰ seawater when fish were transferred to 12‰ or 3‰, but increased by over 100% in the mucus of fish at 50‰, suggesting that it is retained and that it may have a relevant role, perhaps for the activity of immune-related enzymes. To the best of our knowledge, there is no information in the literature regarding alteration of the secretion of skin mucus components, such as ion-binding proteins, during salinity acclimation, but salinity-induced changes in mucus lysozyme activity have been reported in salmonids [103,104].

The changes in ions and osmolality observed in mucus also indicate that the osmotic pressure of the mucus layer may not only buffer the chronic entry of water and loss of ions across the skin at low salinities but that at high salinities, the high concentration of ions in the skin mucus may increase water exudation from the skin which would contribute to increased mucus volume and skin or muscle dehydration. Indeed we showed that fish transferred from 35‰ to 50‰ showed the highest increase in mucus production; however, skin mucus production, either in absolute volume exuded or in relation to fish size, increased when fish were transferred to both hypoosmotic or hyperosmotic conditions. This seems to indicate that the volume of mucus exuded is not only related to simple osmotic gradients but may be regulated. On the contrary, the osmotic parameters of the mucus after 15 days of acclimation to the different salinity conditions were similar to those observed in our previous studies using acute osmotic stress at 3 h [27], suggesting that there are no specific long-term mechanisms to further control skin mucus osmolality. Further studies in protein-osmotic components of skin mucus and how these respond to the hormonal changes that take place during hypo-or hyper-osmotic acclimation will be of great interest to understand better the characteristics, properties and role of skin mucus in fish subjected to salinity challenges. It also remains to be determined if salinity changes the nature of secreted mucins and skin mucus cells, as it happens in gill mucus cells.

### 4.3. Energy Waste by Mucus Exudation

Although skin mucus biomarkers have been considered a powerful tool for determining fish welfare and physiological status via less invasive methods [23,24,25,26,30,37,39,105], there is little work on the use of mucus analysis for the study of long-term chronic stress in fish. In addition to modifying circulating protein concentration, salinity challenges also resulted in important changes in soluble protein exudation into mucus, which significantly increased for the extreme conditions 3‰ and 50‰. This increase begins immediately after transfer, as we previously reported [27], and values are even higher after 15 days of exposure. The reasons for this increase are elusive, since the proteins accounting for it could not yet be identified. It is possible that those may be related to enhanced protection due to the relevance of skin protein components in innate immune defences [31], to higher mucus viscosity [23,25] or to the osmoregulatory properties of skin mucus which may provide a barrier to the movement of ions and water. Lactate exuded in mucus was also greatly affected by salinity, and its concentration was over two-fold higher in the 50‰ condition than in the control group. Although no similar data yet exists in the literature, we recorded the individual volumes of mucus exudation and we were able to calculate the total amount of each biomarker in the mucus. These data could be used to evaluate the energy expenditure and waste required to maintain the considerable amounts of exuded mucus in response to salinity challenges. For the first time, we have demonstrated that these increased amounts of mucus exude large amounts of soluble proteins, lactate and even cortisol, with the 50‰ condition once again causing the greatest effects. Sangiao-Alvarellos et al. [9] reported that the acclimation period is composed of an initial stage of increased energy use (i.e., increases in glucose and lactate) and reorganization of tissue energy metabolism, both in osmoregulatory (gills and kidney) and non-osmoregulatory (liver and brain) tissues. This is followed by the second stage of homeostasis in osmoregulatory parameters and a return to normality of metabolic parameters. Here, we found that energy modification varied mainly in lactate metabolism but also with an important release of protein to mucus, while glucose metabolism was not affected. Thus, we calculated the new steady-stage of sea bass in the hypersaline condition, to be a high energy loss stage compared to control. This is also true for the lowest salinity group, although with a lesser impact, both showed that despite the species euryhalinity there is an important allostatic load to be considered during hyper and hypo-osmotic acclimation. This evidence matches the observations of Boeuf and Payan [50], who measured less energy expenditure for osmoregulation in isosmotic conditions, sparing resources for other physiological processes, such as growth. In addition, mucus exudation showed the same production volume at 50‰ salinity as that of acute stress for 3 h [27], which would mean that fish status was not improved from that of the initial stress being a chronic and putatively harmful condition for the animal. Following the same reasoning, fish maintenance under the 3‰ and 12‰ conditions increased exuded mucus volume similar to those from acute stress [27]. This emphasizes the relevance of skin mucus for coping with salinity acclimation, but also suggest that the initial tolerance of freshwater conditions [27] would result in a chronic challenge in terms of energy expenditure as greater amounts of exuded soluble protein and lactate demonstrate. Whether such fast and long-lasting changes in skin mucus properties are specific for sea bass or differ in species with other degrees of euryhalinity remains to be seen.

## 5. Conclusions

In short, we compared the acclimation of European sea bass one hypoosmotic (3‰), one isosmotic (12‰)and one hyperosmotic (50‰) salinity conditions after 15 days, by measuring morphometric parameters, skin mucus and plasma stress biomarkers, and osmoregulation parameters, together with gill energetic and structural remodeling. Growth was not significantly affected, but a tendency towards decreased growth was noted in extreme conditions (3‰ and 50‰). The volume of skin mucus exuded proved to be an informative parameter: an exacerbated expenditure of energy was recorded in the hypersaline condition, and to a lesser extent in hyposaline conditions, with regard to control values. Gill Na^+^/K^+^-ATPase activity showed a typical “U-shaped” pattern, while gill remodeling resulted in a shift from neutral to acidic mucin mucus cells when moving from hyposaline to hypersaline conditions, together with a decrease in size and an increase in frequency. Skin mucus osmoregulation shifted from facilitating ion capture and ion transport at low salinities to retaining water at high salinities. Herein, we demonstrate the usefulness of skin mucus as a minimally invasive tool to analyze chronic situations, like salinity changes, and the need for further studies of the functions of mucus metabolites.

## Figures and Tables

**Table 1:** Morphometric parameters of European sea bass juveniles submitted to sustained osmotic challenge.

Parameter	3‰	12‰	35‰	50‰
Body weight (g)	118.1 ± 8.5	126.9 ± 9.1	132.2 ± 7.2	122.7 ± 3.7
Body length (cm)	22.25 ± 0.41	22.75 ± 0.46	23.20 ± 0.47	22.95 ± 0.33
Condition factor K	1.06 ± 0.03	1.06 ± 0.02	1.05 ± 0.02	1.05 ± 0.02

Values are shown as mean ± standard error of mean. *n* = 10. No significant differences were shown among salinities challenges (3‰, 12‰, 35‰ and 50 ‰) by one-way ANOVA analysis. 35‰ is assumed as control value of seawater salinity (italic values). Initial body weight (129.2 ± 3.6 g).

**Table 2:** Plasma osmolality and main osmotic-related ions of European sea bass submitted to a chronic osmotic challenge.

Parameter	3‰		12‰		35‰		50‰	
Osmolality (mOsm·Kg^−1^)	322.30 ± 2.88	b	326.50 ± 1.47	ab	338.60 ± 4.5	a	358.40 ± 5.07	c
Sodium (mmol·L^−1^)	161.59 ± 1.26	a	160.22 ± 0.46	a	163.95 ± 1.56	a	171.97 ± 1.75	b
Chloride (mmol·L^−1^)	122.96 ± 8.41	a	129.63 ± 4.97	a	145.99 ± 3.82	ab	159.50 ± 6.33	b
Potassium (mmol·L^−1^)	4.43 ± 0.09	c	4.84 ± 0.07	b	5.20 ± 0.02	a	4.37 ± 0.07	c

Values are shown as mean ± standard error of mean. *n* = 10. Different letters indicate different groups of significance among salinities challenges (3‰, 12‰, 35‰ and 50 ‰) by one-way ANOVA analysis and post-hoc Tuckey’s test (*p* < 0.05). 35‰ is assumed as control value of seawater salinity.

**Table 3:** Skin mucus and plasma biomarkers of European sea bass juveniles submitted to a chronic osmotic challenge.

Mucus Biomarkers	3‰		12‰		35‰		50‰	
Soluble protein (mg/mL)	9.44 ± 0.85	b	7.81 ± 0.84	ab	6.12 ± 0.61	a	10.81 ± 1.05	b
Glucose (μg/mL)	8.41 ± 0.93		6.78 ± 1.11		6.76 ± 1.16		8.71 ± 2.01	
Lactate (μg/mL)	7.94 ± 1.44	a	6.67 ± 1.09	a	6.38 ± 0.79	a	15.53 ± 1.82	b
Cortisol (ng/mL)	1.30 ± 0.25	b	0.89 ± 0.20	ab	0.49 ± 0.21	a	0.45 ± 0.16	a
Plasma biomarkers	**3‰**		**12‰**		**35‰**		**50‰**	
Soluble protein (mg/mL)	25.0 ± 1.1	b	23.3 ± 1.4	ab	15.9 ± 1.3	a	20.1 ± 0.3	a
Glucose (mg/dL)	147 ± 13		124 ± 12		169 ± 21		163 ± 20	
Lactate (mg/dL)	71.7 ± 7.3	b	52.7 ± 2.8	b	39.8 ± 3.1	a	53.7 ± 0.8	b
Cortisol (ng/mL)	225 ± 66	ab	289 ± 132	ab	467 ± 88	a	157 ± 51	b

Values are shown as mean ± standard error of mean. *n* = 10. Different letters indicate different groups of significance among salinities challenges (3‰, 12‰, 35‰ and 50 ‰) by one-way ANOVA analysis and post-hoc Tuckey’s test (*p* < 0.05). The value of 35‰ is assumed as control value of seawater salinity. The relationship for each stress biomarker among plasma and mucus is analyzed by Pearson’s correlations coefficients without any significance.

## Data Availability

Data sharing is not applicable to this article.
